# Undesirable stressful life events, impact, and correlates during midlife: observations from the Seattle midlife women’s health study

**DOI:** 10.1186/s40695-018-0045-y

**Published:** 2019-01-03

**Authors:** Annette Joan Thomas, Ellen Sullivan Mitchell, Nancy Fugate Woods

**Affiliations:** 1 College of Nursing, Seattle University, Seattle, USA; 2 Family and Child Nursing, University of Washington, Seattle, USA; 3 Biobehavioral Nursing and Health Informatics, University of Washington, Seattle, USA

**Keywords:** Stressful life events, Menopausal transition, Midlife women

## Abstract

**Purpose:**

To examine the undesirable stressful life events midlife women experience, including: 1) which life events midlife women reported most frequently; 2) which life events women rated as most undesirable; and 3) whether age, years of education, income, employment, race/ethnicity, marital status, being a parent, and the menopausal transition stage were associated with the impact scores of the life event categories.

**Background:**

In addition to the menopausal transition, midlife is a time of increased responsibilities for women related to multiple roles such as taking care of children, caring for elderly parents, managing households, and working outside the home. These multiple roles put midlife women at risk for increased stress with little time for themselves in order to relieve stress.

**Methods:**

The sample used in this study is part of a larger longitudinal study, The Seattle Midlife Women’s Health Study. Women (*N* = 380 for Occasion 1) completed the 77-item Life Events Scale on four occasions during the course of the SMWHS: Occasion 1 (1990), Occasion 2 (1992), Occasion 3 (1997), and Occasion 4 (2000). In addition to descriptive analyses of frequency of life events and the undesirable impact of life events, demographic correlates (age, education, income, employment, being a parent as well as marital status, race/ethnicity, and menopausal transition stages) were examined in relation to the stressful life event scores.

**Results:**

Highest scores of undesirable life events were for categories of both Financial and Family/Friends over 3 of the 4 occasions. Health and Crime/Legal scores were among the highest for 2 occasions. Impact of the undesirable stressful life events was greatest for categories of Family/Friends; Personal/Social; Work; and, Health. Age, income, marital status, being a parent, and menopausal transition stage were each associated with specific categories of the stressful event impact scores.

**Conclusion:**

Most commonly reported undesirable life events were not those women described as having the greatest impact. Impact of life event stress reflected women’s social roles and connections as seen in the categories with the highest impact scores: Family/Close Friends, Personal/Social, and Work. Menopausal transition stages were related only to undesirable health events.

## Introduction

Although stressful life events have been the focus of research for a variety of populations, there has been relatively little attention paid to understanding the stressful aspects of midlife women’s lives. Many of the studies that have investigated stress in midlife women have used measurements of perceived stress, scales rated at different time points, descriptions, or health-related quality of life; few have used life event scales.

One approach to studying perceived stress among midlife women has been to ask women to rate their stress levels over a period of time, such as the past 24 h, and to relate their ratings to events in their lives, for example progression through the menopausal transition stages. Woods and colleagues [1] examined the relationship of menopausal transition (MT)-related factors (MT stage, urinary estrone glucuronide, follicle-stimulating hormone, aging), psychosocial factors (income adequacy, role burden, social support, parenting, employment, history of sexual abuse, depressed mood) and symptoms (hot flashes, depressed mood, lower sexual desire, difficulty getting to sleep, night-time awakening, early morning awakening, forgetfulness, difficulty concentrating) on perceived stress recorded by Seattle Midlife Women’s Health Study (SMWHS) participants in a daily health diary. They found that perceived stress ratings were unrelated to the menopausal transition stage, but significantly associated with symptoms. Decrease in role burden, social support, and income adequacy was associated with significantly lower levels of perceived stress. Using multivariate models, Freeman and colleagues [2] examined perceived stress on symptoms women experienced during the menopausal transition and found that perceived stress was strongly correlated with symptom severity in the Penn Ovarian Aging Study. Women who reported high stress scores were 40% more likely to report irritability, anxiety, mood swings, concentration difficulties, and headache compared to those women who reported low stress scores [2].

Another approach has been to examine women’s own descriptions of their stressful experiences as they relate to midlife and menopause [3–5]. Woods and Mitchell [3] found that one of the stressful aspects of women’s anticipation of menopause was described as uncertainty about what to expect [5]. Thomas [4] recently reported results of content analysis of women’s descriptions of their most challenging experiences during 15 years of participation in the Seattle Midlife Women’s Health Study, revealing that in addition to challenges involving divorce/breaking up with a partner, death of a parent, and health concerns of their own, a common feature for midlife women was experiencing multiple co-occurring stressors. However, when women looked back over the time period during which they experienced the menopausal transition, menopause did not figure prominently in their most challenging experiences [4].

Other approaches have included researching the impact of stress on modifiable lifestyle factors. To illustrate, in a cross-sectional study from Australia, Seib and colleagues [6] used structural equation modeling to investigate the impact of stress on lifestyle and quality of life using the Life Stressor Checklist-revised (LSC-R; to determine high magnitude stressors) and found that women who reported high magnitude stressors also reported high BMI and more chronic illness. Also, duration of exposure to life stressors was associated with higher depressive symptom scores and sleep disturbance scores [6].

Few studies investigating stress in midlife women utilized life event scales; and, most of these investigations have involved cross-sectional data. For example, in a cross-sectional study from Iran involving women 35 to 55 years, Horri et al. [7] investigated the relationship between education and metabolic syndrome using the stressful life events (SLE) scale and found that the prevalence of metabolic syndrome in women with eight or more SLEs was higher compared to those women who reported fewer than eight SLEs. Furthermore, poorly educated women reported a greater number of SLEs. In a case-control study from New England, Rosman et al. [8] used the Psychiatric Epidemiology Research Interview (PERI) scale to determine the cumulative impact of stressful life events in women on the development of Takotsubo Cardiomyopathy (TC: defined as acute stress-induced cardiomyopathy, a temporary condition where the left ventricle changes shape and the heart becomes suddenly weakened mimicking symptoms of a heart attack) versus women who have had myocardial infarctions (MI) and healthy controls (HC) and found that the death of a relative/close friend and illness/injury to a relative/close friend were more prevalent in women who had TC than those that had a MI or HC. In addition, the onset of TC was associated with multiple SLEs during the 6 months prior to hospitalization suggesting that grief and cumulative stress may play a major role in the onset of Takotsubo Cardiomyopathy [8].

Asking women about their experiences of what researchers have determined are major stressful events, such as a death or an experience of personal injury, is an alternative way to study the stressful aspects of women’s lives. Investigators for the Study of Women and Health Across the Nation (SWAN) explored women’s ratings of life events that were upsetting using the Psychiatric Epidemiology Research Interview (PERI) Life Events Scale, which involved events related to school, work, romantic relations, children, family, crime and legal matters, finances, and health. Hall and colleagues [9] used PERI scores over 9 years to characterize women’s experiences as low, medium, and high levels of chronic stress and related these to sleep [9]. Although SWAN participants rated the degree to which these events were upsetting, researchers have not yet examined the type of stressful events most prevalent in this population.

To date, few studies have focused on the nature of stressful life events midlife women experience, how they view these events, e.g., as undesirable or desirable, and their impact over time as women age. Most early work on stressful life events focused on experiences of men: the original life events scale was developed and used with male naval shipyard personnel [10]. Consequently, experiences that women would rate as stressful were not reflected in any of the earlier stressful life events scales. Norbeck [11] is one of the few investigators to have studied stressful life events among women, having focused on a population of single and married adult women of childbearing age, interviewing them about major events or disruptions that occurred in their lives during the past year and during their preschool child’s first year of life. Norbeck [11] identified stressful events related to contraception, parenting, single-parenting, custody issues, being the victim of assaultive acts, and having difficulty obtaining employment as salient concerns of these women and subsequently created the Life Events Questionnaire (LEQ), a 77-item self-rated scale to assess women’s experiences of life events and their impact, described in greater detail in the methods section. Midlife women have unique concerns, which may or may not differ from those they had during their childbearing years. To date, there have been limited reports of life events experienced by midlife women and how they are appraised and none that characterize how women’s experiences change over time during the menopausal transition. Stressful life events may be related to women’s roles and demographic characteristics that reflect social advantage, but to date these have not been studied.

The Seattle Midlife Women’s Health Study (SMWHS) provides a unique data source that allows for characterizing the patterns of stressful life events examined on four occasions over a ten-year period. These data allow exploration of whether certain types of stressful life events, for example, those related to finances, change over time.

The overall goal of this study was to describe the stressful life events midlife women experience as undesirable and their impact or rating of the event as they progressed through the menopausal transition during the 10 years they were in the SMWHS. The specific aims were to determine 1) which life events midlife women reported most frequently; 2) which events women rated as most undesirable; and 3) whether age, years of education, income, employment, race/ethnicity, marital status, being a parent, and the menopausal transition stages were associated with the impact scores of the life event categories.

## Methods

### Study design and population

Data reported here were from the Seattle Midlife Women’s Health Study (SMWHS), an observational, longitudinal study of approximately 23 years, from 1990 to 2013 [12]. Women entered the study between 1990 and the early part of 1992 when most were in the early stages of the menopausal transition (MT) or not yet in the transition. All households within census tracts with a wide income range and mixed ethnicity were contacted by telephone for interested and eligible women. Women who were eligible were between 35 and 55 years of age, had at least one menstrual period within the last year, had a uterus and at least one ovary, were not pregnant, and could read and understand English. Out of 11,222 telephone contacts, 820 women were eligible, and 508 women entered the study [12].

Women completed an initial in-person interview administered by a trained registered nurse interviewer. A subset of the 508 women kept a health diary and from late 1996 through 2005 provided urine samples. All women were mailed a yearly Health Questionnaire and kept a menstrual calendar.

### Sample

Participants in the current study provided at least one and up to four Life Event Scale (LES) questionnaires beginning in 1990 and who were in either the late reproductive (LR), early transition (ET), late transition (LT) or early post-menopause (PM) sometime during the course of the study. When women started the study, they were on average 42 years, well-educated (16 years of education), earning a gross family income of $35, 740 (SD $15, 440), most were employed (86%), and married (71%). Women identified themselves as African American (12%), Asian/Pacific Islander (9%), White (76%), Latina (1%), and Mixed/Native American (3%). Seventy-five percent of the women were parents (See Table 1).

**Table 1:** Demographic Characteristics of Participants Providing Data for the Life Events Scale for Four Occasions

Characteristic	Occasion 1 *N* = 380	Occasion 2 *N* = 233	Occasion 3 *N* = 220	Occasion 4 *N* = 191
	Mean (SD)	Mean (SD)	Mean (SD)	Mean (SD)
Age	42 (5)	44 (5)	47 (4)	50 (4)
Years of Education	16 (3)	16 (3)	16 (3)	16 (3)
Gross Family Income	35,740 (15,440)	No Data	40,920 (15,042)	43,580 (14,478)
Currently Employed	86%	97%	89%	90%
Race/Ethnicity				
African American	12%	8%	7%	7%
Asian/Pacific Islander	9%	9%	9%	9%
White	76%	81%	84%	84%
Latina	1%	1.5%	1%	0
Mixed/Native Amer	3%	1.5%	0	0
Marital Status				
Never married/partnered	6%	6%	6%	4%
Married/partnered	71%	65%	68%	65%
Divorced/separated	22%	28%	25%	30%
Widowed	2%	1%	2%	1%
If a parent?				
Yes	75%	67%	70%	68%
No	25%	33%	30%	32%
Menopausal Transition				
Stage (MTS), % (N)				
Late Reproduction (LR)	70% (*N* = 142)	58% (100)	43% (66)	38% (47)
Early Transition (ET)	22% (45)	33% (57)	37% (57)	25% (31)
Late Transition (LT)	5% (11)	5% (8)	13% (20)	22% (27)
Post Menopause (PM)	2% (4)	4% (7)	7% (10)	16% (20)

The Life Event Scale (LES) was administered on four occasions: Occasion 1(1990), Occasion 2 (1993), Occasion 3 (1997), and Occasion 4 (2000). Attrition rates for the initial LES during Occasion 1 (1990) included 67 women who were unable to be contacted, five women who became ineligible, and 64 who left the study for personal reasons resulting in 380 women. For the 2nd occasion (1992), thirty-six women were unable to be contacted, a total of 18 women became ineligible, and 32 women left for personal reasons resulting in 233 women. During the third occasion, 18 women were unable to be contacted, ten became ineligible, and 15 who left for personal reasons generating 220 participants. For the fourth and final occasion (2000), nineteen women were unable to be contacted, 138 women became ineligible, and 34 left for personal reasons leaving 191 women.

As seen in Table 1, the mean age of the sample increased at each testing occasion (*F* = 169, *df* = 3, *p* < .001) from 42 years to 50 years, on average, as expected for a longitudinal study. The number of years of education of the women remained the same over time. The participants who remained in the study earned higher incomes over time, possibly reflecting that those with lower incomes tended to leave the study (*F* = 18.602, *df* = 2, *p* < .001). At least 86% of the women were employed as indicated by a higher percentage reporting employment on each occasion and the possibility that those who weren’t employed were more likely to leave the study (Pearson’s Chi Square = 18.864, *df* = 3, *p* < .001). Ethnic composition of the sample also changed over time (Chi Square = 18.907, *df* = 9, *p* = .026) resulting in a higher proportion of White women remaining enrolled with fewer African American, Latina and Mixed/Native American women completing the study. There were no significant differences between occasions for marital status (Pearson’s Chi Square = 7.221, *df* = 9, *p* = .614) although there is a slight decrease in the percentage of married women, suggesting that these women divorced or dropped out of the study and there is a slight increase in the percentage of divorced women over the course of the study. There was a slight decrease, although not statistically significant, in the number of parents who remained in the study (Pearson’s Chi Square = 5.161, *df* = 3, *p* = .160). As expected, there were significant differences among the women in menopausal transition stages across all occasions (Chi square = 81.440, *df* = 9, *p* < .001) with women progressing from the late reproductive (LR) to early (ET) and late (LT) transition and to post-menopause over time.

### Measures

The measures used in this analysis included the Life Events Scale and Menstrual Calendars to determine menopausal transition stage. Demographic characteristics included age, years of education, income, employment, race/ethnicity, marital status, and being a parent.

#### The life events scale

The Life Events Scale (LES*)* is an adaptation of Norbeck’s Life Events Questionnaire (LEQ) [11] created for use with midlife women by the investigators of The Seattle Midlife Women’s Health Study (SMWHS). An enumeration of each item is given in the Appendix. The LES is a 77-item, self-rated scale that assesses whether or not a stressful event happened over the past year and how stressful the event was. The LES was given four times during the course of the SMWHS: Occasion 1 (1990), Occasion 2 (1992), Occasion 3 (1997), and Occasion 4 (2000). The LES has the same nine sections or categories as the LEQ: Health, Work, Residence, Love & Marriage, Family & Close Friends, Parenting, Personal & Social, Financial, and Crime & Legal Matters.

The LES differs from the LEQ in the wording of the questions seen in the categories of Work; Family and Close Friends; and, Personal and Social. The following items were changed to reflect relevance to midlife. In the Work category, one item, “job changed,” was added. For Family and Close Friends, “death of parents” and “birth of a grandchild” were added; and “acquired or lost a pet” was omitted. The questions were adapted in item 5a (see items listed in Appendix) to include “grandchild” and in item 5 g to include “other family members (than a parent).” In the Personal and Social category, one item was added, “lost a friend for other reasons.” In summary, after the adaptations for midlife women, both the LEQ and the LES totaled 77 items.

Women were asked to indicate whether or not a life event had occurred over the past year (yes/no); to evaluate if the event was undesirable, neutral or desirable; and, to rate the impact of the event as 1) no effect, 2) small effect, 3) moderate effect, or 4) great effect. For this investigation, undesirable mean total scores and undesirable total impact scores were reported for all 9 sections of the LES as well as the totals for the individual undesirable items under each section over 4 separate occasions spanning 10 years.

#### Menopausal transition stages

Menopausal Transition Stages (MTS) were labeled according to the stages of reproductive aging developed by Mitchell, Woods, and Mariella [13]: Late reproductive stage (LR), early menopausal transition (ET), late menopausal transition (LT), or post-menopause (PM), and match those labels recommended at the Stages of Reproductive Aging Workshop (STRAW) [14], and STRAW + 10 [15–17]. The data were obtained from menstrual calendars and coded as LR, ET, LT, or PM based on criteria developed by the Seattle Midlife Women’s Health Study (SMWHS) [13] and validated by Harlow and colleagues [17, 18]. The late reproductive (LR) stage includes the time in midlife before the onset of persistent menstrual cycle irregularity when cycles are regular; Early transition (ET) stage is defined as persistent irregularity of more than 6 days of absolute difference between the start of any two consecutive menstrual cycles during the year, with no skipped periods; Late transition (LT) is defined as persistent skipping of one or more menstrual periods. A skipped period was defined as 60 or more consecutive days of amenorrhea during the calendar year [18]. Persistence indicated the irregular cycle or skipped period took place one or more times in the ensuing 12 months. The final menstrual period (FMP) was identified retrospectively after 1 year of amenorrhea. The first day of the FMP was used to determine age of onset of the FMP. Early post menopause (PM) was the time frame within 5 years after the FMP.

#### Demographic characteristics

Age was measured in years, as was education. Income from all sources was measured as gross family monthly pay in dollars. Current employment (part time or full time) was assessed using employed or not employed. Ethnicity was self-reported as African American, Asian/Pacific Islander, White, Latina, or Mixed/Native American. Marital status was self-reported as never married/never partnered, married/partnered, divorced/separated, or widowed. Parental status was assessed by asking women whether or not they were parents, including parenting adopted and foster children (See Table 1).

##### Data analysis

Descriptive statistics (mean, standard deviation) were used to describe the individual items of the Life Event Scale (LES), scale scores, estimates of undesirable impact of the LES and demographic variables by occasion when the questionnaires were administered. To assess the relationship between demographic factors (age, years of education, gross family income, employment, race/ethnicity, marital status, being a parent) and life event stress, Pearson’s *r* was used for continuous variables and analysis of variance was used for categorical variables (menopausal transition stage, race/ethnicity). Descriptive statistics, Pearson’s *r*, and analyses of variance were performed using SPSS v23.

## Results

### Frequency of undesirable events

The total scores (number of items reported, standard deviation, N, adjusted means) of undesirable events for each category of the Life Event Scale rated over the past year are presented in Table 2. The total scores were calculated to identify which categories had the highest number of undesirable events. Adjusted mean total scores represent the simple average number of items for each of the subscales, e.g., health, work, etc., calculated by dividing the total number of items selected by the total number of items in each subscale to enable comparison across subscales, e.g., health vs. work.

**Table 2:** Number of all *Undesirable* Events by LES Categories^a^ by Occasion (Mean N of Items Reported (SD), Adjusted Mean)

Category (Number of Items)	Occasion *N* = 381	Occasion *N* = 235	Occasion 3 *N* = 221	Occasion 4 *N* = 192
Health (9)	.73 (.99) .08	.48 (.79) .05	.42 (.73) .05	.42 (.76) .05
Work (15)	.64 (1.00) .04	.69 (1.00) .05	.48 (.92) .03	.54 (.95) .04
Residence (4)	.18 (.46) .05	.09 (.31) .02	.05 (.22) .01	.07 (.31) .02
Love & Marriage (13)	.71 (1.20) .05	.50 (.98) .11^b^	.38 (.86) .03	.31 (.70) .02
Family & Close Friends (8)	.72 (.82) .09	.69 (.81) .04	.49 (.71) .06^b^	.51 (.72) .06^b^
Personal & Social (12)	.75 (1.03) .06	.60 (.81) .05	.56 (.84) .05	.58 (.88) .05
Financial (5)	.48 (.72) .10^b^	.29 (.55) .06	.32 (.66) .06^b^	.25 (.49) .05
Crime & Legal Matters (6)	.42 (.65) .07	.37 (.63) .06	.29 (.59) .05	.30 (.59) .05
Parenting (5)	.40 (.69) .08	.26 (.54) .05	.18 (.50) .04	.15 (.44) .04
Total Number of Undesirable Events	5.02 (3.82)	3.98 (2.71)	3.18 (2.97)	3.13 (3.01)

^a^ Adjusted mean total scores represent the simple average number of items for each of the subscales, calculated by dividing the total number of items selected per category by the number of items in each category to enable comparison across categories

^b^ Represents the highest category per Occasion

Overall categories with the highest adjusted total scores for undesirable events were: Financial; Love and Marriage; and, Family and Close Friends. The categories with the highest adjusted total scores varied over the years of the study. For Occasion 1, the largest adjusted mean total number of stressful events by category were: Financial (.10), Family and close Friends (.09), Health (.08), and Parenting (.08). For Occasion 2, the largest adjusted mean total number of stressful events were in the categories of Love and Marriage (.11), Financial (.06), and Crime/Legal (.06). For Occasion 3, Family and Friends (.06) and Financial (.06) categories followed by Health, Personal/Social, Crime/Legal Matters (.05 each) were the largest adjusted mean total number of stressful events and for Occasion 4, the largest adjusted mean total number of events were in the categories of Family and Friends (.06), Health, Personal/Social, Financial, and Crime/Legal Matters (.05 each).

To summarize, over the four occasions, the adjusted mean total for the Financial category was among the highest, followed by Family and Friends for three of the four occasions. Health/Parenting and Crime/legal categories had the highest adjusted total means for Occasions 1 and 2, respectively. Thus, some categories of the most frequently experienced life events changed over the occasions of the study.

### Mean impact scores of undesirable events

Over all occasions, the highest rated undesirable impact scores by category included Family and Close Friends. Personal/Social, Health and Work categories also had high impact scores. As seen in Table 3, the LES categories with the highest mean impact scores for undesirable events for Occasion 1 included Family & Close Friends (1.75), followed by Personal/Social (1.49) and Health (1.41). The categories of Family/Close Friends (1.65), Work (1.29), and Personal/Social (1.28) had the highest impact scores for Occasion 2. Similarly, the categories of Family/ Close Friends (1.32), Personal/ Social (1.12) and Work (.96) had the highest mean impact scores for Occasion 3. For the last Occasion, the highest impact scores were Family/ Close Friends (1.36), Personal/Social (1.21), and Work (1.03). Both Occasion 3 and 4 followed the same order of highest impact scores. Of note is that the lowest undesirable impact scores for each occasion were for the Residence category.

**Table 3:** Mean Impact Scores of *Undesirable* Events by LES Categories by Occasion [Mean impact score (SD)]

Category (No. items)	Occasion 1 *N* = 381	Occasion 2 *N* = 235	Occasion 3 *N* = 221	Occasion 4 *N* = 192
Health (9)	1.41 (1.63)	1.05 (1.49)	.92 (1.45)	.88 (1.43)
Work (15)	1.31 (1.66)	1.29 (1.58)	.96 (1.50)	1.03 (1.50)
Residence (4)	.50 (1.23)	.28 (.91)	.16 (.70)	.21 (.86)
Love & Marriage (13)	1.30 (1.67)	.96 (1.52)	.75 (1.41)	.69 (1.37)
Family & Friends (8)	1.75 (1.76)^a^	1.65 (1.73)^a^	1.32 (1.72)^a^	1.36 (1.74)^a^
Personal & Social (12)	1.49 (1.64)	1.28 (1.52)	1.12 (1.49)	1.21 (1.56)
Financial (5)	1.20 (1.66)	.77 (1.38)	.77 (1.44)	.70 (1.36)
Crime & Legal Matters (6)	.96 (1.41)	.86 (1.41)	.66 (1.27)	.63 (1.21)
Parenting (5)	.90 (1.45)	.59 (1.20)	.42 (1.08)	.36 (1.00)

An average impact score was calculated by dividing the total of impact ratings by the number of women who reported the items to give the average impact score

^a^ Depicts the highest impact score per occasion

### Individual items of each category

Analysis of individual items (see Appendix) provided further clarification of which specific items of the categories of life events were most commonly rated and viewed as having the most undesirable impact. The impact scores were rated as 1) no impact, 2) small impact, 3) moderate impact or 4) great impact. An average impact score was calculated by dividing the total of impact ratings by the number of women who reported the items to give the average impact score. The percentage calculated was the total number of women who reported the event, divided by the number of women for that particular occasion.

The most frequently reported individual items in the Health category included a major change in eating habits or major dental work. The most frequently reported Work items were having changed work hours or conditions, changed responsibilities at work, and having troubles at work with an employer or co-worker. Although Residence was the category with the lowest score totals for undesirable events (see Tables 2 and 3), the most frequently reported individual item was having had a major change in living conditions, such as a home improvement or a decline in home or neighborhood. There were two most frequently reported items in the Love and Marriage category: having had a change in closeness with a husband or life partner and having had a change in a husband or partner’s work outside the home. The most frequently reported Family and Close Friends items included having gained a new family member; experiencing a major change in the health or behavior of a family member or close friend; and, experiencing the death of a family member or close friend. The most frequently reported Personal and Social events included having had a vacation; had a trip, not a vacation; and, made a new friend. Most frequently reported Financial concerns involved having had a major change in financial status, improved or worsened, followed by taking on a moderate purchase, such as a car or major appliance. Crime and Legal Matters events were less frequently reported and included being involved in a minor violation of the law, getting traffic tickets or arrested for disturbing the peace, being robbed, or being involved in a car accident. The most frequently reported Parenting events included conflicts with a husband or partner about parenting and a change in childcare arrangements.

### Associations of undesirable impact scores with demographic characteristics

Table 4 includes the correlations among the undesirable impact scores of the LES with baseline measures (Occasion 1) of demographic characteristics. Age was significantly correlated with undesirable impact scores for health (*r* = 0.103, *p* = .046): older women had higher undesirable impact scores for health-related events. Women with higher income reported lower undesirable impact scores for: health events (*r* = −.133, *p* = .010), events concerning residence (*r* = −.115, *p* = .025), love and marriage events (*r* = −.133, *p* = .010) and financial matters (*r* = −.192, *p* < .001). Being a parent was associated with reporting a higher undesirable impact of financial events (*r* = .102, *p* = .046) as well as parenting life event stress, as expected (*r* = .259, *p* < .001). Years of education and employment status were not significantly correlated with any of the undesirable impact scores for the LES categories.

**Table 4:** Correlation Matrix of Impact Scores of Undesirable Events at Baseline (Occasion 1) and Demographic Categories (Pearson’s *r*, *p* value, and N for each)

LES subscale/ correlate	Health	Work	Residence	Love and Marriage	Family and Close Friends	Personal and Social	Financial	Crime and Legal Matters	Parenting
Age	0.103* .046 (380)	−.058 .258 (380)	.004 .943 (380)	.003 .946 (380)	.033 .515 (380)	.060 .244 (380)	−.064 .217 (379)	.074 .151 (380)	−.063 .224 (380)
Years of Education	−.072 .163 (380)	.086 .095 (380)	−.027 .602 (380)	.007 .889 (380)	−.063 .222 (380)	−.010 .854 (380)	−.023 .655 (379)	.066 .200 (380)	−.013 .795 (380)
Income	−.133** .010 (375)	−.027 .608 (375)	−.115* .025 (375)	−.133** .010 (375)	−.097 .060 (375)	−.087 .094 (375)	−.192** .000 (374)	−.035 .500 (375)	−.082 .115 (375)
Employment	−.081 .114 (380)	.044 .391 (380)	−.041 .421 (380)	−.087 .089 (380)	.017 .746 (380)	−.066 .196 (380)	−.050 .336 (379)	−.041 .430 (380)	−.028 .583 (380)
If a parent	.046 .368 (380)	−.041 .420 (380)	.018 .727 (380)	−.010 .841 (380)	.009 .859 (380)	.014 .786 (380)	.102* .046 (379)	−.031 .542 (380)	.259** .000 (380)

*r* = Pearson’s Correlation

*p* is significant if *: α < .05 or **: α < .001

*N* = Number of women

Marital status, race/ethnicity, and menopausal transition stages were also examined for their association with undesirable total impact scores for LES categories using analysis of variance. For the first occasion, marital status was associated significantly with greater undesirable impact scores for Health (*F* = 3.109; 3, 376 df; *p* = .026), Love and Marriage (*F* = 6.979; 3, 376 df; *p* < .001), and Personal and Social events (*F* = 3.920; 3, 376 df; *p* < .001). Post Hoc analysis identified a significant negative mean difference between Married/Partnered and Divorced women indicating that being married was associated with less impact of stressful health events than being divorced. Being married also was associated with a lesser impact of stressful events in Love and Marriage compared to women who were never married and to women who were divorced. Married women reported less impact of Personal and Social stressful life events than did women who were divorced.

For the second occasion, undesirable impact scores of Love and Marriage (*F* = 7.430; 3, 229 df; *p* < .001) as well as Personal and Social events (*F* = 2.770; 3, 229 df; *p* = .042) were associated with marital status. Married women reported significantly more of an impact of undesirable stressful life events in Love and Marriage than divorced women, while divorced women reported a greater impact of stressful life events in Love and Marriage than women who had never been married. Married women reported a greater impact of undesirable stressful life events in the Personal and Social category than women who were widowed. Marital status was not associated with the LES undesirable mean total impact scores for the third occasion.

For Occasion 4, four categories were associated with marital status: Work (*F* = 4.231; 3, 187 df; *p* = .006), Residence (*F* = 3.209; 3, 187 df; *p* = .024), Personal/Social (*F* = 3.417; 3, 187 df; *p* = .019), and Financial/Legal Matters (*F* = 2.971; 3, 187 df; *p* = .033). Women who were never married rated the impact of Work stress and Personal and Social stress significantly higher than married women. Married women rated impact scores significantly higher than divorced women for Work, Residence, Personal and Social, and Financial stress. Married women also reported significantly higher impact scores of Financial stress than women who were widowed.

Ethnicity was significantly associated with the undesirable impact scores for the Financial category, but only for Occasion 2 (F = 3.879; 4, 228 df; *p* = .005) and Occasion 3 (F = 7.772; 3, 216 df; *p* < .001). For Occasion 2, there were significant differences between Hispanic/Latina women and all other ethnicities suggesting that Hispanic/Latina women rated the impact of Financial stress higher than women who were Asian/Pacific Islander, African American, White and Mixed/Native American; however, only three women identified themselves as Hispanic/Latina. Post hoc analyses were not performed for Occasion 3 because at least one group had fewer than two cases. One woman identified herself as Hispanic/Latina for the third occasion and no women identified themselves as Mixed/Native American.

Menopausal transition stages were significantly associated with undesirable mean impact scores only for Health and only for Occasion 1 (*F* = 3.700; 3, 198 df; *p* = .013), but not significant for all other occasions. Significant mean differences from the Post Hoc analysis tests from the analysis of variance for the LES category impact scores for undesirable stressful life events and the menopausal transition stages (LR = Late Reproductive, ET = Early Transition, LT = Late Transition, PM = Post-menopausal) were evident for Occasion 1. Scores for the LR, ET, and LT stages each significantly and negatively differed from the PM stage (*p* < .001, .001, and .008) in the Health category, meaning that the mean impact scores of undesirable Health events decrease as women transition through the menopausal stages; however, there were only four women in the post-menopausal stage during the first occasion.

## Discussion

The overall goal of this study was to describe the undesirable life events midlife women experience, women’s ratings of the impact of these life events, and factors associated with the experience of stressful life events by category. Over 10 years of follow-up, participants in the Seattle Midlife Women’s Health Study reported the highest adjusted mean total score of undesirable life events in the Financial category, followed by Family and Close Friends for three of the four occasions. Health and Crime/Legal categories were among the highest adjusted mean total scores for two occasions. In contrast, the highest mean *impact* scores for undesirable life events over all four occasions were in the category of Family and Close Friends. Thus, the most commonly reported undesirable life events (financial) may not have been those women described as having the greatest impact (family and close friends).

Women’s assessment of the impact of life events reflected their social roles and connections as shaping the kinds of stressful life events they experienced and their impact, as seen in the categories with the highest impact scores of Family/Close Friends, Personal/Social, and Work. Indeed, investigators have recently identified the importance of women’s roles and relationships in relation to the types of stressors they experience. Woods-Giscombé et al. [19] differentiated self-stress and network-stress among African American women aged 21 to 78 years. Self-stress referred to stressful events happening to oneself whereas network-stress referred to stressful events happening to family, friends and loved ones, which could indirectly affect the woman herself. African American women reported significantly more network-stress than self-stress [19]. Certainly, Family and Close Friends as well as Personal and Social life events could reflect network-stress as described by Woods-Giscombé et al. [19]. Midlife women’s social roles and connections thus may place them at risk for a greater number of stressors altogether as indicated by events related to their networks as well as those that they experience directly. These findings also support the utility of the concept of self in relation, as described by Jean Baker-Miller, whose conception of women’s development emphasizes the importance of life experienced in a relational network [20]. Understanding the life experiences women rate as undesirable and high impact requires viewing their experiences from their individual perspectives as they reflect their place in a relational network.

In addition to describing the number and impact of life events in the various categories, the undesirable impact of life events was correlated with several demographic factors. Mean impact scores for undesirable stressful life events were associated with age, not surprising given that one of the categories with the largest number of undesirable stressful life events during Occasion 1 was Health. In fact, women reported a major change in eating habits (29%), a major change in recreation (25%), a major change in sleeping habits (23%), and a major personal illness or injury (22%), all with a moderate to great impact score (See Appendix). Undesirable impact scores for Health, Residence, Love/Marriage, and Financial life events were also correlated with family income, reflecting the effects of social disadvantage on the experience and impact of stressful events. Although education was not correlated with undesirable mean impact scores, the current results are similar to what others have found. Prior studies have examined how social structure is linked to health in midlife and younger women 25–64 years of age. McDonough et al. [21] found that chronic stressors (social life, financial, relationship, child, environment, and family health stress) were significantly and positively related to women’s levels of distress and that health status improved as education and household income increased. Furthermore, in the current study, married women reported lower impacts of Health stress than did women who were divorced, results supported by McDonough et al. [21] who revealed that married women reported the best health results while formerly married women reported the worst. Newton et al. [22] investigated midlife women aged 51 to 60 years and found that married women experienced fewer functional limitations and fewer risks of chronic diseases (hypertension, diabetes, heart attacks, chronic heart failure, coronary heart disease, angina, stroke and rheumatoid arthritis) compared to women who were divorced/separated, widowed and never married.

Family income was also associated with the undesirable impact of life events in the Financial category. Women with lower family incomes may have been less able to bear the costs of purchasing and maintaining a residence, and may have had difficulty paying for food, bills or rent. In addition, lower income may place a strain on Love and Marriage; one of the most frequently reported Love and Marriage items was a change in husband or partner’s work outside the home (26%) see Appendix.

Being a parent was associated with more Financial stress, such as paying for a child’s food and clothing, as well as Parenting stress. Women most frequently reported having a change in childcare arrangements (23%) and conflicts with their husband or partner about parenting (31%) see Appendix.

### Strengths and limitations

One of the strengths of this study was that chronic life event stress was investigated in midlife women repeatedly over a decade. Life event stress may reflect the impact of the chronic activation (stress arousal) of the hypothalamic-pituitary-adrenal (HPA) axis. Future investigations may consider how, and if, desirable or positive life events buffer the effects of negative or undesirable stressful life events on HPA axis responses, such as cortisol levels.

Among the limitations of this study was the declining number of participants over time reflecting the attrition that occurs during longitudinal studies and women becoming ineligible due to having completed early post-menopause as well as other causes such as use of hormone therapy. Also, exploring the influence of factors beyond the demographic variables was beyond the scope of this paper. For example, depressed mood is an important factor that both influences one’s perception of life event stress and may be a consequence of a stressful life event. In the current study, 202 of the 380 women who completed the Occasion1 LES, completed a Center for Epidemiologic Studies Depression Scale (CESD) resulting in a mean sum score of 11.4, median score of 10.4, and a SD 7.3. These results indicate that the women who completed the CESD in the current study had a depression score less than 16; the literature suggests a sum score of 16 or more as an indicator of depression. In the longitudinal Study of Women’s Health Across the Nation (SWAN), women who experienced more upsetting life events were more likely to experience depressed mood [23, 24]. Future studies of stressful life events of midlife women should include factors such as depression in order to determine whether women with depressed mood identify more stressful life events and rate them as having a greater undesirable impact in addition to determining whether the LES scores predict depressed mood. Also, future studies of patterns of change or continuity over time in life event stress would be insightful for clinicians to help midlife women manage their life event stress.

## Conclusion

Midlife women experience a variety of stressful life events with a range of undesirable impact of these events. The types of events and their impact were related to women’s roles as well as to factors that afforded them social advantage, such as income. In addition, there were uniform patterns of decline over time of the impact of undesirable life events suggesting that adaptation may occur over time as well as the possible loss of participants experiencing the most stressful events.

Two important messages from these results follow. First, practitioners will benefit from knowing the type of stressful life events occurring for women, especially those related to their roles and social advantage during midlife, a time of many different responsibilities. Second, many women have multiple co-occurring stressors, potentially related to an initiating event, such as divorce. Divorce may propagate additional stressful events, such as loss of income and/or insurance, the need to increase hours worked, difficulty with childcare arrangements, as well as loss of a partner. Undesirable stressful life events that continue over time may place women at risk for development of pathologies such as hypertension, diabetes, heart disease, arthritis, and obesity. More research is needed to understand the potential consequences of this snowballing effect that often happens in midlife women’s lives.

## Appendix

**Table 5:** Number of Women (And Percent of Total for each Occasion) Reporting Individual LES Items and Undesirable (U) Impact Scores (Mean, SD) for each Occasion

Item	Occasion 1 (% of total)	U	Occasion 2	U	Occasion 3	U	Occasion 4	U
1. Health subscale	N = 381		N = 235		N = 221		N = 192	
*A. major* personal illness or injury	83 (22%)	3.33 (.83)	36 (15%)	3.5 (.72)	43 (20%)	3.5 (.76)	31 (16%)	3.25 (.74)
*B. major* change in eating habits	111 (29%)	4.0 (.00)	56 (24%)	3.0 (1.00)	46 (21%)	4.00 (ND)	38 (20%)	4.00 (ND)
*C. major* change in sleeping habits	89 (23%)	3.8 (.45)	32 (14%)	3.4 (.55)	45 (20%)	3.0 (ND)	32 (17%)	2.0 (ND)
*D. major* change in usual type and/or amount of recreation	94 (25%)	4.0 (0.00)	56 (24%)	3.5 (.71)	42 (19%)	3.5 (.71)	29 (15%)	3.0 (ND)
*E. major* dental work	65 (17%)	3.0 (ND)	72 (31%)	4.0 (ND)	24 (11%)	3.0 (ND)	17 (9%)	4.0 (ND)
*F. major* difficulty with birth control pills or devices	12 (3%)	4.0 (ND)	3 (1%)	4.0 (ND)	5 (2%)	2.0 (ND)	4 (2%)	3.0 (ND)
g. Pregnancy	21 (6%)	4.0 (ND)	7 (3%)	ND	2 (1%)	ND	0 (0%)	ND
h. miscarriage or abortion	8 (2%)	3.0 (ND)	4 (2%)	4.0 (ND)	0(0%)	ND	0(0%)	ND
i. started menopause	47 (12%)	3.0 (ND)	26 (11%)	4.0 (ND)	31 (14%)	4.0 (ND)	35 (18%)	4.0 (ND)
2. Work								
a. had difficulty finding a job	36 (9%)	4.0 (ND)	27 (12%)	4.0 (ND)	13 (6%)	3.0 (ND)	8 (4%)	4.0 (ND)
b. begun work outside the home	62 (16%)	4.0 (ND)	15 (6%)	3.0 (ND)	15 (7%)	4.0 (ND)	8 (4%)	4.0 (ND)
c. changed job setting, but continued the same kind of work	97 (26%)	4.0 (ND)	47 (20%)	2.0 (ND)	37 (17%)	3.0 (ND)	43 (22%)	3.0 (ND)
d. changed to a new type of work	72 (19%)	4.0 (ND)	35 (15%)	4.0 (ND)	32 (15%)	4.0 (ND)	27 (14%)	4.0 (ND)
e. changed work hours or conditions	161 (42%)	3.5 (.71)	91 (39%)	3.0 (ND)	78 (35%)	4.0 (0.00)	63 (33%)	2.0 (ND)
f. changed responsibilities at work.	138 (36%)	4.0 (ND)	96 (41%)	4.0 (ND)	67 (30%)	4.0 (ND)	53 (28%)	4.0 (ND)
g. had troubles at work with employer or co-workers	128 (34%)	3.5 (.58)	69 (29%)	4.0 (ND)	61 (28%)	4.0 (ND)	40 (21%)	3.0 (ND)
h. had a major business readjustment	46 (12%)	4.0 (ND)	27 (12%)	4.0 (ND)	24 (11%)	4.0 (ND)	14 (7%)	4.0 (ND)
i. been fired or laid off from work	26 (7%)	4.0 (ND)	20 (9%)	4.0	8 (4%)	4.0	10 (5%)	4.0 (ND)
j. retired from work	5 (1%)	4.0 (ND)	3 (1%)	ND	0	ND	3 (2%)	ND
k. started courses by mail or studying at home to help with work	41 (11%)	4.0 (ND)	13 (6%)	ND (ND)	14 (6%)	3.0 (ND)	6 (3%)	3.0 (ND)
l. begun or ended school, college, or training program	65 (17%)	3.0 (ND)	32 (14%)	4.0 (ND)	19 (9%)	ND	12 (6%)	4.0 (ND)
m. changed career goal or academic major	54 (14%)	4.0 (ND)	19 (8%)	ND	25 (11%)	3.0 (ND)	12 (6%)	4.0 (ND)
n. changed school, college or training program	10 (3%)	ND	6 (3%)	2.0 (ND)	4 (2%)	4.0 (ND)	3 (2%)	4.0 (ND)
o. had problems in school, college or training program	9 (2%)	4.0 (ND)	10 (4%)	4.0 (ND)	3 (1%)	2.0 (ND)	1 (< 1%)	3.0 (ND)
3. Residence								
a. had difficulty finding a home	18 (5%)	3.58 (.67)	3 (1%)	2.5 (.71)	3 (1%)	3.0 (ND)	3 (2%)	3.67 (.58)
b. changed residences within the same town or city	39 (10%)	3.13 (1.13)	14 (6%)	4.0 (0.00)	6 (3%)	ND	9 (5%)	4.0 (ND)
c. moved to a different town, city, state, or country	15 (4%)	2.67 (1.21)	14 (6%)	4.0 (0.00)	6 (3%)	ND	4 (2%)	ND
d. had a major change in living conditions (home improvements or a decline in home or neighborhood)	121 (32%)	3.26 (.85)	55 (23%)	2.94 (.77)	44 (20%)	3.10 (.88)	34 (18%)	3.67 (.50)
4. Love and Marriage								
a. begun a new, close personal, romantic relationship	40 (11%)	3.33 (.58)	23 (10%)	3.50 (.71)	22 (10%)	4.0 (ND)	17 (9%)	4.0 (ND)
b. become engaged	20 (5%)	ND	7 (3%)	ND	4 (2%)	ND	4 (2%)	4.0 (ND)
c. had girlfriend or boyfriend problems (not just friends)(not husband/partner)	48 (13%)	3.43 (.69)	28 (12%)	3.55 (.67)	19 (9%)	3.13 (.83)	12 (6%)	3.33 (.87)
d. broken up with a boyfriend (or girlfriend) or broken an engagement	46 (12%)	3.52 (.80)	20 (9%)	3.58 (.52)	16 (7%)	3.18 (.87)	5 (3%)	4.0 (0.00)
e. gotten married or begun to live with someone (roommate OK)	37 (10%)	3.5 (.58)	15 (6%)	ND	11 (5%)	3.5 (.58)	12 (6%)	ND
f. had change in closeness with husband or life partner	140 (37%)	3.36 (.73)	63 (27%)	3.27 (.83)	42 (19%)	3.28 (.90)	37 (19%)	3.13 (.89)
g. experienced infidelity, (cheating on husband/partner)(either party)	33 (9%)	3.48 (.73)	16 (7%)	3.55 (.69)	12 (5%)	3.83 (.41)	8 (4%)	3.2 (1.30)
h. had trouble with in-laws	36 (9%)	3.07 (.73)	11 (5%)	2.6 (.84)	14 (6%)	2.73 (.91)	8 (4%)	3.25 (.89)
i. separated from husband or life partner due to conflict	29 (7%)	3.67 (.77)	10 (4%)	3.50 (.84)	11 (5%)	4.0 (0.00)	8 (4%)	4.0 (0.00)
j. separated from husband or life partner due to work, travel, school, etc.	36 (9%)	3.17 (.99)	15 (6%)	3.0 (0.00)	10 (5%)	3.25 (.96)	4 (2%)	3.33 (1.16)
k. had a reconciliation with spouse or partner	25 (7%)	ND	10 (4%)	3.50 (.71)	11 (5%)	ND	4 (2%)	ND
l. had a legal divorce	12 (3%)	4.0 (0.00)	3 (1%)	ND	2 (< 1%)	ND	3 (2%)	4.0 (0.00)
m. had a change in husband’s or partner’s work outside the home (beginning work, ceasing work, changing jobs, retirement, etc.)	100 (26%)	3.48 (.72)	43 (18%)	3.35 (.70)	36 (16%)	3.46 (.69)	35 (18%)	3.00 (.54)
5. Family and Close Friends								
a. gained a new family member (through birth, adoption, relative moving in, includes extended family)	109 (29%)	2.8 (1.03)	51 (22%)	3.67 (.52)	40 (18%)	3.00 (.82)	47 (25%)	3.0 (1.00)
b. had a child or family member leave home (due to marriage, to attend college, or for some other reason)	59 (16%)	3.48 (.81)	27 (12%)	3.9 (.32)	37 (17%)	3.63 (.74)	28 (15%)	3.75 (.50)
c. had a major change in the health or behavior of a family member or close friend (illness, accidents, drug or disciplinary problems, etc.)	165 (43%)	3.41 (.75)	100 (43%)	3.28 (.80)	75 (34%)	3.42 (.72)	79 (41%)	3.55 (.59)
d. had the death of a husband or partner	4 (1%)	4.0 (0.00)	1 (< 1%)	4.0 (ND)	1 (< 1%)	4.0 (ND)	2 (1%)	2.0 (ND)
e. had the death of a child	1 (< 1%)	4.0 (ND)	1 (< 1%)	ND	0	ND	1 (< 1%)	ND
f. had the death of a parent	36 (9%)	3.92 (.27)	19 (8%)	3.79 (.43)	13 (6%)	3.4 (.70)	8 (4%)	3.67 (.82)
g. had the death of another family member or close friend	103 (27%)	3.30 (.78)	60 (26%)	3.22 (.76)	44 (20%)	3.2 (.76)	35 (18%)	3.29 (.64)
h. had a change in the marital status of your parents	8 (2%)	3.5 (.58)	5 (2%)	3.5 (.71)	4 (2%)	4.0 (0.00)	1 (< 1%)	ND
6. Personal and Social								
a. major personal achievement	139 (37%)	3.0 (1.41)	70 (30%)	ND	52 (24%)	4.0 (ND)	48 (25%)	3.0 (1.41)
b. had a major decision regarding the immediate future	154 (40%)	3.62 (.51)	70 (30%)	3.56 (.88)	67 (30%)	3.40 (.89)	55 (29%)	3.88 (.35)
c.								
d. had a change in political beliefs	8 (2%)	3.50 (.71)	5 (2%)	ND	5 (2%)	ND	3 (2%)	ND
e. had a change in religious beliefs	20 (5%)	3.50 (.71)	10 (4%)	ND	15 (7%)	ND	4 (2%)	ND
f. had a loss or damage of personal property	75 (20%)	3.12 (.83)	41 (17%)	2.97 (.81)	49 (22%)	2.81 (.83)	24 (13%)	3.00 (.79)
g. had a vacation	231 (61%)	3.55 (.69)	151 (64%)	3.00 (ND)	163 (74%)	3.00 (1.00)	125 (65%)	4.00 (0.00)
h. had a trip; not a vacation	171 (45%)	3.15 (.99)	98 (42%)	2.72 (.91)	107 (48%)	3.11 (.93)	96 (50%)	3.00 (.82)
i. had a change in family get-togethers	91 (24%)	3.17 (.80)	60 (26%)	2.80 (.78)	75 (34%)	3.04 (.77)	54 (28%)	3.09 (.81)
j. had a change in social activities (clubs, movies, visiting)	102 (27%)	2.76 (.74)	66 (28%)	2.84 (.60)	56 (25%)	2.78 (.81)	43 (22%)	3.07 (.73)
k. made a new friend	231 (61%)	2.25 (.50)	123 (52%)	4.0 (ND)	100 (45%)	ND	95 (50%)	3.50 (.71)
l. broken up with a friend due to conflict	66 (17%)	3.18 (.83)	30 (13%)	2.93 (.70)	28 (13%)	3.20 (.78)	21 (11%)	3.33 (.72)
m. lost a friend for other reasons (death, moving)	72 (19%)	3.30 (.79)	47 (20%)	2.94 (.69)	25 (11%)	2.80 (.86)	35 (18%)	2.75 (.85)
7. Financial								
a. had a major change in financial status (improved or worsened)	168 (44%)	3.53 (.68)	92 (39%)	3.33 (.76)	79 (36%)	3.58 (.50)	69 (36%)	3.20 (.76)
b. taken on a moderate purchase such as a car, major appliance, etc.	157 (41%)	2.79 (.98)	85 (36%)	2.92 (.90)	70 (32%)	3.0 (.93)	59 (31%)	2.75 (.96)
c. taken on a major purchase or a mortgage loan, such as a home, business, property, etc.	67 (18%)	3.29 (.83)	40 (17%)	2.50 (.71)	39 (18%)	3.0 (.78)	30 (16%)	3.40 (.55)
d. experienced a foreclosure on a mortgage or loan	7 (2%)	3.17 (.98)	0	ND	1 (< 1%)	4.0 (ND)	1 (< 1%)	3.0 (ND)
e. had credit rating difficulties	75 (20%)	3.26 (.70)	23 (10%)	2.84 (.69)	19 (9%)	3.38 (.89)	10 (5%)	3.25 (.71)
8. Crime and Legal Matters								
a. robbed	49 (13%)	3.22 (.84)	22 (9%)	3.28 (.75)	26 (12%)	2.82 (.67)	11 (6%)	2.64 (.51)
b. a victim of a violent act (rape, assault, etc.)	3 (1%)	4.0 (0.00)	4 (2%)	3.75 (.50)	4 (2%)	3.33 (1.16)	2 (1%)	3.0 (ND)
c. involved in a car accident	45 (12%)	2.74 (.91)	31 (13%)	2.89 (.97)	27 (12%)	2.90 (.79)	29 (15%)	2.77 (.87)
d. involved in a law suit	35 (9%)	3.05 (.83)	14 (6%)	2.73 (1.01)	16 (7%)	3.80 (.45)	15 (8%)	3.0 (1.00)
e. involved in a minor violation of the law (traffic tickets, disturbing the peace, etc.)	75 (20%)	2.15 (.69)	37 (16%)	2.40 (.91)	30 (14%)	2.54 (.78)	23 (12%)	2.21 (1.12)
f. involved in legal troubles resulting in your being arrested or held in jail	0	ND	2 (1%)	4.0 (ND)	1 (< 1%)	3.0 (ND)	0	ND
9. Parenting								
a. had a change in child care arrangements	88 (23%)	3.11 (.90)	34 (15%)	3.60 (.89)	23 (10%)	2.86 (.90)	17 (9%)	3.0 (.82)
b. had conflicts with husband or partner about parenting	119 (31%)	3.13 (.84)	51 (22%)	2.68 (.85)	37 (17%)	2.86 (.83)	26 (14%)	2.74 (.87)
c. had conflicts with child’s grandparents (or other important person) about parenting	40 (11%)	2.87 (.78)	17 (7%)	2.46 (.82)	10 (5%)	3.29 (.76)	5 (3%)	4.0 (0.00)
d. taken on full responsibility for parenting as a single parent	37 (10%)	3.0 (.93)	10 (4%)	3.67 (.58)	8 (4%)	4.0 (ND)	9 (5%)	4.0 (0.00)
e. custody battles with former husband or partner	6 (2%)	3.60 (.89)	2 (1%)	3.0 (ND)	3 (1%)	3.0 (1.41)	1 (< 1%)	4.0 (ND)
